# *In vivo* Calcium Imaging Reveals That Cortisol Treatment Reduces the Number of Place Cells in Thy1-GCaMP6f Transgenic Mice

**DOI:** 10.3389/fnins.2019.00176

**Published:** 2019-03-01

**Authors:** Tim Indersmitten, Michael J. Schachter, Stephanie Young, Natalie Welty, Stephani Otte, Jonathan J. Nassi, Timothy Lovenberg, Pascal Bonaventure, Ryan M. Wyatt

**Affiliations:** ^1^Janssen Research & Development, LLC., San Diego, CA, United States; ^2^Inscopix, Palo Alto, CA, United States

**Keywords:** place cells, cortisol, citalopram, calcium imaging, miniature microscopes, GCaMP6f, transgenic mice

## Abstract

The hippocampus, a structure essential for spatial navigation and memory undergoes anatomical and functional changes during chronic stress. Here, we investigate the effects of chronic stress on the ability of place cells to encode the neural representation of a linear track. To model physiological conditions of chronic stress on hippocampal function, transgenic mice expressing the genetically encoded calcium indicator GCaMP6f in CA1 pyramidal neurons were chronically administered with 40 μg/ml of cortisol for 8 weeks. Cortisol-treated mice exhibited symptoms typically observed during chronic stress, including diminished reward seeking behavior and reduced adrenal gland and spleen weights. *In vivo* imaging of hippocampal cellular activity during linear track running behavior revealed a reduced number of cells that could be recruited to encode spatial position, despite an unchanged overall number of active cells, in cortisol-treated mice. The properties of the remaining place cells that could be recruited to encode spatial information, however, was unperturbed. Bayesian decoders trained to estimate the mouse’s position on the track using single neuron activity data demonstrated reduced performance in a cue richness-dependent fashion in cortisol-treated animals. The performance of decoders utilizing data from the entire neuronal ensemble was unaffected by cortisol treatment. Finally, to test the hypothesis that an antidepressant drug could prevent the effects of cortisol, we orally administered a group of mice with 10 mg/kg citalopram during cortisol administration. Citalopram prevented the cortisol-induced decrease in single-neuron decoder performance but failed to significantly prevent anhedonia and the cortisol-induced reduction in the proportion place cells. The dysfunction observed at the single-neuron level indicates that chronic stress may impair the ability of the hippocampus to encode individual neural representations of the mouse’s spatial position, a function pivotal to forming an accurate navigational map of the mouse’s external environment; however, the hippocampal ensemble as a whole is resilient to any cortisol-induced insults to single neuronal place cell function on the linear track.

## Introduction

Chronic stress has a profound impact on the pathophysiology of psychiatric disorders and shares a number of clinical features associated with anxiety and mood disorders. Symptoms of depression include changes in the hypothalamic–pituitary–adrenal (HPA) system, which manifest as altered regulation of corticotropin and cortisol secretory activity, as well as elevated glucocorticoid levels (Holsboer, 2000; Vreeburg et al., 2009). The effect of glucocorticoids on the pathophysiology of depression is well characterized. For example, subjects administered with corticosteroids exhibit changes in sleep EEG typically observed in depression (Antonijevic and Steiger, 2003). Additionally, in both chronic stress and depression, elevated corticosteroid levels are associated with neuronal atrophy and loss of synaptic spines in cortical and limbic brain regions (Krishnan and Nestler, 2008; Savitz et al., 2010; Duman, 2014). Corticosteroid-induced pathophysiological changes also cause cognitive and emotional symptoms in depressed patients, such as memory deficits and decreased motivation and reward seeking behavior. Importantly, impaired HPA axis function in patients suffering from depression is successfully treated with antidepressants (Holsboer and Barden, 1996; Holsboer, 2000; Raison and Miller, 2003; Pariante et al., 2004).

The fact that stress is a significant factor in the development of major depressive disorder serves as the rationale for studying experimental models of stress and depression. Similar to patients suffering from depression, plasma corticosterone levels are elevated in mouse models of depression (Gómez-Lázaro et al., 2011). Additionally, corticosterone administration reliably increases depression-like behaviors in rodents in a time and dose-dependent manner (Kalynchuk et al., 2004; Gregus et al., 2005; Brummelte et al., 2006; Johnson et al., 2006; Marks et al., 2009). Mice administered with corticosterone exhibit immobility during forced swimming, anhedonia during sucrose preference and urine sniffing, impaired sexual and reward behavior, and impaired spatial working memory (Gorzalka et al., 1998, 2003; Sousa et al., 2000; Coburn-Litvak et al., 2003; Hill et al., 2003; Gourley et al., 2008; Murray et al., 2008). Glucocorticoid receptors are abundantly expressed in the mouse hippocampus (Wang et al., 2013), and treatment with corticosterone inhibits hippocampal neurogenesis and volume, an effect that can be rescued by treatment with antidepressants (Murray et al., 2008; David et al., 2009). Thus, the close relationship between corticosterone levels and depression-like symptoms in mice is a useful tool for modeling depression. Advantages of corticosteroid administration are the high construct and predictive validity, good control of standardized dosing, the fact that endogenous corticosterone levels can be easily manipulated by corticosteroid administration, and most importantly the inability of mice to habituate to the stressor even after several months of administration (McLay et al., 1998).

Prior studies have examined the effects of stress on spatial memory, synaptic plasticity and single hippocampal place cell function with the Morris Water Maze, brain slice field potential recordings and multielectrode recordings, respectively (Kim et al., 2007; Park et al., 2015). Importantly, there is still little insight into how stress affects place cell function on the ensemble level during the execution of a behavioral task. Here, we addressed this question by studying how chronic cortisol administration affects place cell function *in vivo* during linear track running using miniaturized microscopy in mice expressing the genetically encoded calcium sensor GCaMP6f. Additionally, we co-administered the antidepressant citalopram with cortisol to test the hypothesis that citalopram could rescue the effect of chronic cortisol treatment, since citalopram has been shown to alleviate behavioral symptoms of chronic stress related to motivation and reward sensitivity (Rygula et al., 2006; Araya-Callis et al., 2012). We found that cortisol causes stress-related changes in physiology and behavior and led to depressive-like symptoms. Compared to untreated mice, cortisol treatment reduced the proportion of active place cells as well as the performance of a Bayesian decoder trained to predict mouse location from neuronal activity.

## Materials and Methods

### Mice and Surgeries

All procedures were approved by the Janssen Research & Development Institutional Animal Care and Use Committee and were performed in accordance with the Guide for the Care and Use of Laboratory Animals (United States National Institutes of Health). Male transgenic GP5.17 mice (Dana et al., 2014; The Jackson Laboratory, Bar Harbor, ME, United States) age 3.4 ± 0.3 months (mean ± SEM) underwent a 1-h surgery under 1.5–2.0% isoflurane and 0.05 mg/kg Buprenex as described in Berdyyeva et al. (2014). Briefly, a 3 mm diameter portion of the skull was removed with a trephine drill bit at stereotactic coordinates -2.3 mm anterio-posterior, 1.89 mm medio-lateral relative to bregma. 30 gauge blunt needles were used to aspirate the cortex and corpus callosum over the CA1 region of the hippocampus, after which a guide cannula (Inscopix, Palo Alto, CA, United States) was implanted to a depth of 1.09 mm. During surgery, the exposed tissue was constantly rinsed with pH-buffered Ringer’s solution. The cannula was sealed in place with Metabond (Parkell, Edgewood, NY, United States) and dental cement and covered with Kwik Cast (World Precision Instruments, Sarasota, FL, United States). Two weeks after recovery from surgery, a microendoscope GRIN lens (Inscopix, Palo Alto, CA, United States) was secured within the guide cannula using ultraviolet-curing adhesive (Norland, NOA81, Edmund Optics, Barrington, NJ, United States). Finally, a miniature microscope baseplate (Inscopix, Palo Alto, CA, United States) was attached with dental cement under 1.5–2.0% Isoflurane anesthesia.

### Experimental Timeline

**FIGURE 1:**
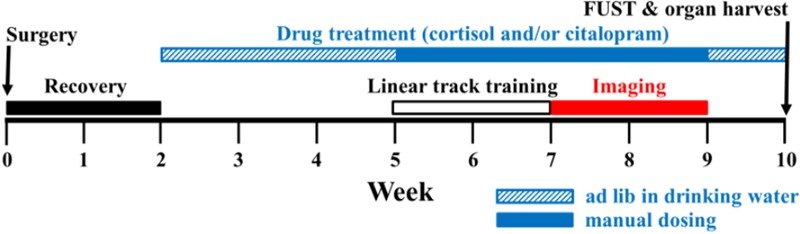
Experimental timeline. Timeline of the experimental design. Treatment groups consisted of mice that were administered either cortisol only, cortisol with citalopram, citalopram only, or control vehicles. All four treatment groups had an *n* = 6 mice. After cannulation surgery, mice were allowed to recover for 2 weeks before drug treatment was initiated (weeks 0–2). To ensure effects of cortisol treatment were sufficient to model conditions of chronic stress, a 3-week period of drug treatment was maintained prior to exposing the mice to the linear track (weeks 2–5). Following 2 weeks of training on the linear track (weeks 5–7), mice were imaged during a 2-week period of daily linear track running (weeks 7–9). Mice recovered from water restriction for 1 week (weeks 9–10) before female urine sniffing test (FUST) administration and organ harvest. Cortisol (40 μg/ml) was added into the drinking water during *ad lib* water consumption (weeks 2–5 and 10) or administered subcutaneously (10 mg/kg) (weeks 5–9). Likewise, citalopram (10 mg/kg) was administered daily by oral gavage (weeks 2–5 and 10) and injected subcutaneously (10 mg/kg) during weeks 5–9. Untreated mice received drinking water or subcutaneous saline injections during water restriction sessions.

To create a mouse model of chronic stress in which place cell function could be measured, we followed the experimental timeline shown in Figure 1. Following the cannulation surgery, mice were divided into four treatment groups of six mice each: cortisol (cort), cortisol + citalopram (cort + cit), citalopram, or untreated. Cortisol (given in their drinking water) and citalopram (oral administration) treatment started after a 2-week post-surgical recovery period. To avoid confounding effects of acute vs. chronic stress on formation of place fields, drugs were administered to mice for 3 weeks prior to training on the linear track. During the training period, mice were gradually water-restricted to 1 ml/day and habituated to the linear track. Two weeks of training were followed by two additional weeks of water restriction and linear track running, during which place cell activity was recorded with miniature microscopes (nVistaHD; Inscopix, Palo Alto, CA, United States). After conclusion of the imaging experiments, drug treatment continued for one additional week while animals were allowed to recover from water restriction. In total, drug administration was continuous for a duration of 8 weeks. At the end of this period, mice were tested in the female urine sniffing test (FUST) and organs were harvested to assess the efficacy of cortisol treatment on behavior and physiology.

### Drug Administration

To model physiological effects of chronic stress, we divided mice into four separate treatment groups. Mice in the untreated group received either standard mouse drinking water or subcutaneous saline injections during water restriction. Mice in the cortisol group received 40 μg/ml cortisol in the form of water-soluble hydrocortisone (Sigma-Aldrich, St. Louis, MO, United States) during *ad lib* water consumption (weeks 2–5 and 10) and 10 mg/kg cortisol subcutaneously during water restriction (weeks 5–9). To evaluate effects of a selective serotonin reuptake inhibitor, a separate group of cortisol-treated mice were also orally gavaged daily with 10 mg/kg citalopram hydrobromide (Sigma-Aldrich, St. Louis, MO, United States) during *ad lib* water consumption and injected subcutaneously with 10 mg/kg citalopram hydrobromide during water restriction. Finally, a fourth group of mice only received 10 mg/kg citalopram hydrobromide.

### Linear Track and Behavioral Setup

To record place cells in the dorsal hippocampus with miniature microscopes, we modeled the experimental design after the methods used by Ziv et al. (2013). Mice were gradually water restricted to 1 ml/day and trained to run back and forth on a custom-made 76 cm linear track for 20 min per day for 2 weeks, excluding weekends. To facilitate context discrimination, the left half of the linear track featured rich contextual tactile and visual cues, whereas the right half featured plain contextual cues. Overhead lights were dimmed by red sleeves and two 30 lumens desk lamps and an overhead IR-light illuminated the setup.

### Linear Track Running Sessions

Before each session, miniature microscopes were mounted under light anesthesia. Miniature microscope cables were suspended over the linear track by a custom-built pulley system. After 30 min of recovery from anesthesia inside the home cage, mice were trained to run on the linear track by dispensing 10 μl liquid rewards of mouse drinking water through modified liquid dippers (Med Associates, Fairfax, VT, United States) at each end of the linear track. Automated liquid reward dispensing was controlled by mouse behavior via EthoVision XT software (Noldus, Leesburg, VA, United States). If the mouse traversed the full length of the linear track and entered the reward zone at one of the ends of the track, a liquid reward was dispensed on the opposite end of the track, yielding one liquid reward for each linear track traversal. Total daily reward volume consumption was between 0.5 and 1.5 ml/day. Mice were supplemented by subcutaneous saline injections to ensure a minimum of 1 ml/day fluid consumption and maintenance of at least 80% of initial body weight.

### Recording Sessions

Following training sessions, we imaged dorsal hippocampal CA1 pyramidal neurons expressing GCaMP6f daily for a 2-week period. A GigE 33GP1300 camera (The Imaging Source, LLC, Charlotte, NC, United States) was synchronized with the microscope to record mouse position on the linear track. To assure correct synchronization of frame rate acquisition, the output signal of the microscope was recorded with WinEDR Strathclyde Electrophysiology software (University of Strathclyde). We used nVista software (Inscopix, Inc.) to control image acquisition with miniature microscopes. The microscope LED power was set between 50 and 80% of maximum and a gain setting of 1 was used to maximize GCaMP6f fluorescence signal to noise ratio, while avoiding signal clipping. Image resolution was 1080 × 1440 pixels. We limited the image acquisition rate to 10 Hz to compensate for the lower fluorescence signal of the GCaMP6f expressing neurons in GP5.17 mice compared to virally expressed GCaMP6f. Before the first imaging session, the turret of the microscope was adjusted vertically to a field of view that exhibited the most active neurons with a strong signal. After determining the ideal imaging plane, the turret was fastened with a set screw and not re-adjusted again. Each miniature microscope was dedicated to one specific mouse during its 2-week imaging period (Figure 1) to ensure constant imaging parameters across sessions. Before each recording session, a snapshot was taken and compared to snapshots from previous recording sessions to assure that the mounting position of the miniature microscopes was consistent. Recording sessions during which mice traversed the entirety of the linear track less than 36 times were excluded. For a more detailed protocol about surgeries and linear track training, see Indersmitten et al. (2018).

### Assessment of Cortisol Treatment Effects on Behavior and Physiology

After the last recording session, mice were taken off the water restriction regimen, while cortisol and citalopram treatment continued via drinking water or oral gavage, respectively. After 1 week of recovery from water restriction, anhedonia was assessed using the FUST. Urine was collected and combined from 4 to 6 C57Bl/6 females to ensure that at least one mouse was in the reproductive estrous cycle. After 30 min of habituation, an experimenter blinded to the treatment group presented the male mice with two Q-tips, one soaked in female urine and the other in water. Cumulative time spent sniffing urine and water was recorded for 3 min. Following the FUST, spleen, adrenals, and brains were harvested, although not from all mice. Brains were fixed in 10% neutral buffered formalin solution for at least 24 h and stored in 30% sucrose and 0.02 sodium azide at 4°C for cryosectioning to verify correct microendoscope placement.

### Data Analysis

Images acquired by the miniature microscopes were processed with Mosaic software (Inscopix, Palo Alto, CA, United States) to correct for motion artifacts and identify individual cells in the recording field, from which calcium transients can be extracted and calcium events can be detected (Resendez et al., 2016). An example of this process is illustrated in Supplementary Figure 1. For a given animal, images from multiple recording sessions were spatially down-sampled by a factor of four, concatenated sequentially, registered to correct for motion artifacts, and transformed to relative changes in fluorescence (Δ*F*/*F*). Principal component analysis (PCA) and independent component analysis (ICA) were used to extract neurons and their corresponding calcium traces (Mukamel et al., 2009). Individual components were accepted or rejected manually. Accepted calcium traces were low pass filtered at 1 Hz. Calcium events of neurons were detected using changes in Δ*F*/*F* with a minimal size of 9 units of median absolute deviation (MAD) and a minimal decay time constant (τ) of 150 ms. Each trace was divided by its MAD, and the derivative of the trace was monitored for positivity. When the derivative of the trace was positive and the value of the trace crossed the threshold of 9, the trace was then monitored to ensure that it decayed to a value that was greater than or equal to exp(-t_peak_/τ) within time period t_peak_ + τ, where t_peak_ was the time of peak amplitude for the event. If these criteria were fulfilled, an event was identified with a time of occurrence equal to t_peak_. The peak amplitude was also recorded. To verify that cell identity was preserved over multiple recording sessions for active cells, we sampled a subset of data to quantify the magnitude of centroid shift displacement errors for cell maps registered across recording sessions.

Extracted calcium event data was then analyzed with custom written software in MATLAB (Mathworks, Natick, MA, United States) to identify place cells and measure place field properties. To analyze place fields, mouse position on the linear track was detected by dynamic subtraction of the background image and pixel smoothing using EthoVision XT software. Epochs in which mice ran slower than 3 cm/sec were discarded. We divided the linear track into 20 bins of 3.6 cm and discarded the two most lateral bins on each end of the linear track, in which consummatory behavior of liquid rewards occurred. We then determined the time spent in each bin and normalized the counted frequency of calcium events for bin occupancy. The maximum event rate was normalized to 1. We calculated spatial information in bits/event for all neurons as described in Skaggs et al. (1993) and Rubin et al. (2015):

$$\text{Spatial}\;\mathrm{Information}=\sum_{\text{i}}^{\text{bins}}{\text{p}}_{\text{i}}{\text{(r}}_{\text{i}}\text{/}\bar{r}){\text{log}}_{\text{2}}\left({\text{r}}_{\text{i}}\text{/}\bar{r}\right)$$

where *p_i_* is the probability of the mouse being in bin *i, r_i_* is the event rate for the neuron in bin *i*, and 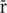 is the overall spike rate. We determined statistical significance using a shuffling approach. For each cell, the event times with respect to the track location was permuted 1,000 times and the spatial information was recomputed for each permutation. This produced a distribution of shuffled spatial information. A lower bound for statistical significance was set as the 99th percentile of the shuffled spatial information distribution. If a neuron’s non-shuffled spatial information exceeded this, it was declared a place cell. All other neurons were defined as non-place cells. Resulting place cell tuning curves for left and right running directions were smoothed with a Gaussian filter (σ = 2.86 bins). Place field position was defined by its location of peak activity and place cells were sorted according to place field position on the linear track.

Spatial selectivity was calculated by returning the common logarithm of the infield event rate divided by the outfield event rate for each individual tuning curve. Spatial selectivity was then averaged for all place cells within a mouse and again for all mice in a treatment group. The infield for a place cell is defined as the linear track bins that correspond to the full width at half of the cell’s maximum event rate. Bins that were not a member of the infield constituted the outfield. To determine the in-and outfield event rates, the event rates for all bins that are members of the in- and outfields, respectively, were averaged for all place cells and recording sessions. The common logarithm was then calculated over this average for each running direction, and then averaged for running direction.

Spatial stability was calculated by measuring the shift between the location of a cell’s place field peak on the first recording session and its place field peak on subsequent recording sessions. The place field peak is defined as the bin with maximal event rate on a particular recording session. To calculate spatial precision, the bin with maximal event count was determined for each place cell during the first recording session. The mean centroid shift for all place cells was then calculated between the first recording sessions and subsequent recording sessions. Mean centroid shift was converted to cm and normalized to 1. A value of 1 indicates that no centroid shift, with tuning curve peaks of subsequent recording sessions being in the same bin as in the first recording session.

To determine place field size, the bins with the half-max and half-min event rate with respect to the highest event rate (tuning curve peak) was determined. The distance between the half-max and half-min bins was then calculated in cm.

### Decoders

We predicted the mouse position by training ensemble and single neuron decoders. We trained empirical Bayes classifiers to predict discretized spatial location on the linear track from ensemble activity, similar to Ziv et al. (2013). Ensemble activity was represented as a binary matrix of size *N* ×*T*, where *N* was equal to the number of neurons and *T* was equal to the total number of frames in the recording session. The frame rate of the movies was 10 Hz and so each time bin was 100 ms long. Element *X*_it_ of the ensemble activity matrix was equal to 1 if neuron i had a calcium event peak in time bin *t*, and was zero otherwise. The discretized spatial location was represented as a categorical variable *y_t_* that could take on a value from the set {1,…, 16}, each element representing one of the 16 spatial bins. For each time bin *t*, we constructed a feature vector *f_t_* from the matrix *X* that included not just the binary ensemble activity at time *t*, but the ensemble activity in the preceding 500 ms (five bins) as well. The feature vector *f_t_* was set to:

$$f_{t}=flatten\left(X_{:,\text{t-5}:\text{t}}\right)$$

where the colon notation X_:,t-5:t_ means to take all rows of *X* and 5 columns, spanning from column *t*-5 to column *t*. To flatten the resulting matrix means to concatenate all the rows into one long vector. The resulting feature vector *f_t_* has a length of 5N.

A Naive Bayes classifier was used to predict the categorical spatial bin *y_t_* from the feature vector *f_t_*, referred to as the “decoder.” The MATLAB function fitncb() was used with the output variable specified as multinomial. The distribution of ensemble activity within a recording session was assumed to be stationary over time. The classifier was only trained on time bins belonging to successful runs. A spatial trajectory was determined to be a successful run if the mouse ran from one end zone on the linear track to the other end zone, and the ratio of forward movements to backward movements exceeded 2. The ensemble decoder was trained on 20 randomly selected successful runs. Separate decoders were trained on left vs. right running directions. The validation error was estimated on the remaining runs, by computing the median absolute error of the predicted spatial bin position in centimeters, minus the actual spatial bin position in centimeters. Each ensemble decoder was trained on data for a recording session in a single day.

The single neuron decoder was trained in a similar manner as the ensemble decoder with two differences. First, for the single neuron decoder, the matrix *X* only had one row, corresponding to the binary calcium event activity of a single neuron. Second, considerable variability was observed in the validation error for the single neuron decoder, so 10 randomly selected training/validation sets were trained on. The average median absolute error across the 10 trained decoders was reported for the single neuron decoder error.

### Statistical Analysis

Adrenal glands, spleen weights and number of neurons were analyzed with one-way ANOVAs. FUST data were analyzed with a linear mixed effect model using R (R Core Team, 2018) and lme4 (Bates et al., 2015) as follows: cumulative sniffing time was modeled as a function of treatment group × liquid type (water vs. urine). Intercept within mouse was included as random factor. Linear track running (number of linear track runs and running velocity) was analyzed with mixed effects model and Tukey tests for multiple comparisons. Treatment group and day of recording session were fixed factors and intercept within mouse was included as random factor.

Treatment with cortisol and citalopram had strong effects on running behavior and the number of linear track runs within an imaging session. To rule out that the treatment group differences in linear track running affected data volume and quality when computing the place cell measures (proportion, event rate and amplitude, selectivity, stability and field size of place cells), we truncated recording sessions after 50 linear track runs to equalize the number of linear track runs for all treatment groups. We chose to include the first 50 runs, since mice traversed the linear track at least 50 times for 96% of recording sessions.

The logit-transformed proportion of place cells was analyzed as a function of treatment group with a logistic mixed effects model to evaluate if drug treatment was a significant predictor on the number of place cells during the first 50 runs. Likewise, event rate and amplitude, selectivity, stability and field size of place cells during the first 50 runs were analyzed with linear mixed effect models in the same fashion. Recording session + treatment groups served as fixed factors and intercept within mouse as random factor in our mixed effects models. Linear mixed effects models with recording session × treatment groups were performed to determine interaction effects between predictors, and linear mixed effect models with recording session:treatment group fixed factors were run to determine the effects of elapsed recording sessions on treatment groups. Tukey tests were performed for multiple comparisons. Given the multitude of hypotheses tested with mixed effects models, all *p*-values were adjusted for multiple comparisons with the Bonferroni correction method using p.adjust in R. We used ks.test to compare centroid shift distributions between treatment groups. Single and ensemble decoder results were analyzed with mixed effects with neuron effects nested in mouse effects.

## Results

### Cortisol Treatment Induces Stress-Like Changes in Physiology and Behavior

**FIGURE 2:**
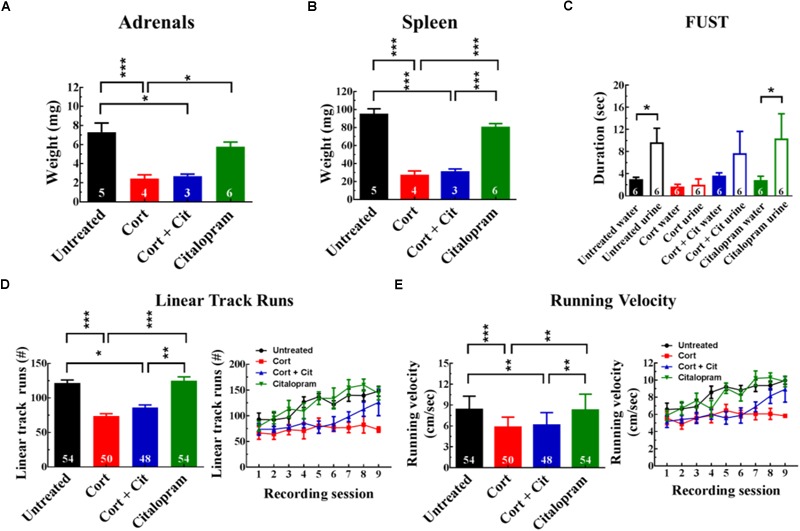
Cortisol treatment induced stress-like changes in physiology and behavior. **(A,B)** Organs were harvested and weighed after 8 weeks of drug treatment. Cortisol-treated mice and cortisol + citalopram-treated mice had significantly reduced adrenal (sum of left and right adrenals) and spleen weights compared to untreated and citalopram-treated mice. Numbers in bars on the graphs indicate the number of animals used. Note that adrenals and spleens were not harvested from all mice. **(C)** Female urine sniffing test administered to mice following chronic drug treatment. Untreated and citalopram-treated mice exhibited urine sniffing preferences. Chronic cortisol treatment eliminated this preference, which was not rescued by citalopram co-administration. **(D)** Linear track runs averaged over all recording sessions (left). Cortisol-treated mice and cortisol + citalopram-treated mice had significantly fewer linear track runs compared to untreated and citalopram-treated mice. Daily linear track runs plotted over time (right). A total of six mice per treatment group, with nine recording sessions per mouse, for a maximal N of 54 were used. **(E)** Average velocity during linear track running (left) and running velocity plotted over time for individual recording sessions (right). Cortisol-treated mice showed significantly reduced running velocity compared to untreated mice, an effect that was not reversed with citalopram co-administration. Cort, cortisol; Cit, citalopram. Asterisks show group comparisons with ^∗^*p* < 0.05, ^∗∗^*p* < 0.01, and ^∗∗∗^*p* < 0.005.

Measurements of organ weight following chronic cortisol treatment showed that cortisol causes stress-related changes in physiology (Figure 2A,B). One-way ANOVAs with treatment as an independent variable and adrenal gland or spleen weight as dependent variables found significant treatment group effects. Cortisol-treated mice had a three times lower weight for adrenals compared to untreated mice (untreated = 7.30 ± 2.16 mg [mean ± SEM]; cort = 2.43 ± 0.81 mg, *p* < 0.005) and for spleen weight (untreated = 94.38 ± 14.20 mg; cort = 26.78 ± 10.07 mg, *p* < 0.005). Co-administration of citalopram with cortisol did not normalize organ weight, as cortisol + citalopram-treated mice also had significantly lowered adrenal weight (cort + cit = 2.70 ± 0.36 mg, *p* < 0.05) and spleen weight (cort + cit = 30.53 ± 6.09 mg, *p* < 0.005) compared to untreated mice. Note that co-administration of citalopram was not expected to prevent reduction in organ weight.

In addition to the effect on physiology, cortisol-treated mice exhibited diminished urine sniffing, a depressive-like symptom, during FUST administration (Figure 2C). A mixed effects model found that cortisol-treated mice exhibited no preference for sniffing urine over water. Although statistical power of test and sample were limited because of small group sizes (*n* = 6), untreated mice sniffed urine significantly longer than water (untreated: urine – water, β = 6.67 seconds, *SE* = 3.33, *p* < 0.05) [β, estimated difference; SE, standard error; see Table 1 for estimated differences between treatment groups]. Citalopram did not prevent loss of urine sniffing preference in cortisol-treated mice, as there was no significant difference in urine vs. water sniffing duration for cortisol + citalopram-treated mice.

**Table 1:** Mixed-effects model estimates.

Measure	Group comparison	Estimated differences	Standard error	*p*-Value
Female urine sniffing test (sec)	Untreated: water - urine	-6.667	3.33	<0.05
	Cort: water - urine	-0.333	3.33	0.920
	Cit: water - urine	-4.000	3.33	0.230
	Cit: water - urine	-7.500	3.33	<0.05
Linear track runs (number)	Untreated - cort	44.788	12.85	<0.001
	Untreated - cort + cit	32.936	12.86	0.010
	Cit - cort	48.121	12.85	<0.001
	Cit - cort + cit	36.270	12.86	0.004
Running velocity (cm/sec)	Untreated - cort	2.385	0.71	<0.001
	Untreated - cort + cit	2.092	0.72	0.003
	Cit - cort	2.310	0.72	0.001
	Cit - cort + cit	2.012	0.72	0.004
Proportion place cells ( 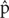 ) (left running direction)	Untreated - cort	0.440	0.16	0.033
	Untreated - cort + cit	0.416	0.21	0.308
	Cit - cort	0.237	0.21	1.000
	Cit - cort + cit	0.099	0.21	1.000
Proportion place cells ( 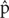 ) (right running direction)	Untreated - cort	0.480	0.17	0.032
	Untreated - cort + cit	0.220	0.17	0.000
	Cit - cort	0.677	0.17	<0.001
	Cit - cort + cit	0.419	0.17	0.093
Single neuron decoder prediction error (cm) (left running direction)	Untreated - cort	-1.210	2.02	0.549
	Untreated - cort + cit	2.145	2.02	0.287
	Cit - cort	-5.115	2.05	0.012
	Cit - cort + cit	-1.760	2.03	0.386
Single neuron decoder prediction error (cm) (right running direction)	Untreated - cort	-3.013	1.52	0.046
	Untreated - cort + cit	-0.599	1.47	0.684
	Cit - cort	-5.397	1.55	<0.001
	Cit - cort + cit	-2.983	1.51	0.047

Linear track running behavior of water-restricted mice trained to obtain liquid rewards at either end of the linear track was analyzed with mixed effects models. Cortisol-treated mice (74 ± 3.74 runs) [mean ± SEM] traversed the linear track significantly fewer times than untreated mice (122 ± 4.42 runs; *p* < 0.001) during the recording session (Figure 2D). Citalopram failed to prevent the reduction of linear track runs in mice administered with cortisol, as cortisol + citalopram-treated mice (86 ± 3.88 runs, *p* = 0.01) also had significantly fewer linear track runs compared to untreated mice. Citalopram alone (125 ± 5.64 runs) did not significantly change the number of linear track runs when compared to the untreated controls. In addition, the mixed effects model found a significant effect of recording session on the number of linear track runs (Figure 2D, right panel). Linear track runs increased significantly with subsequent recording sessions for untreated mice (*p* < 0.001), cort + cit-treated mice (*p* < 0.001) and citalopram-treated mice (*p* < 0.001), but not for cort-treated mice. Furthermore, there was a significant treatment group × recording session interaction effect, with linear track runs increasing significantly less in cort-treated mice compared to untreated mice (*p* = 0.003). In contrast, the increase in runs from mice treated with cort + cit and citalopram was not significantly different from untreated mice.

Cortisol affected running velocity in a similar fashion as linear track runs (Figure 2E). Untreated mice (8.38 ± 5.64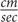 ) had significantly higher running velocity compared to cortisol-treated mice (5.81 ± 0.21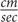 ; *p* < 0.001) and mice co-administrated with cortisol and citalopram (6.12 ± 0.26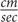; *p* = 0.003). Running velocity increased significantly with recording sessions for all groups (untreated: *p* < 0.001; cort: *p* = 0.025; cort + cit: *p* < 0.001; cit: *p* < 0.001). Like linear track runs, running velocity increased significantly less in cort mice compared to untreated mice (*p* = 0.002).

### Place Cell Imaging in Thy1-GCaMP6f Transgenic Mice With Miniature Microscopes

**FIGURE 3:**
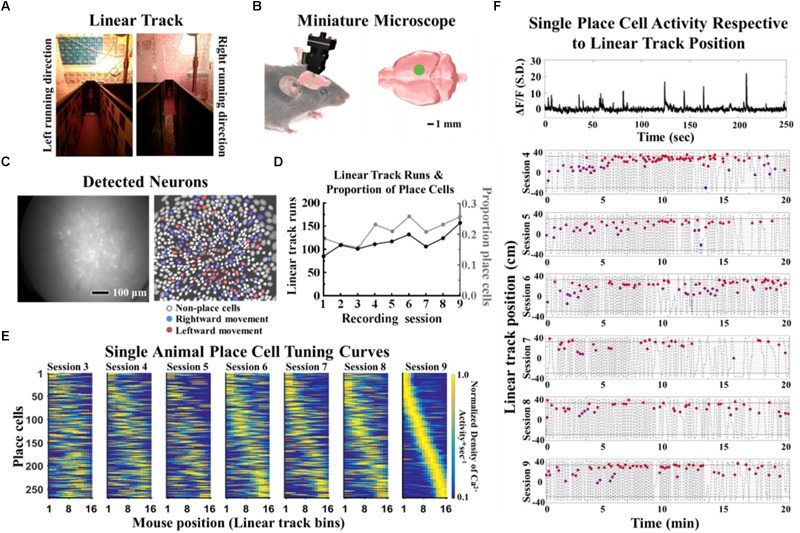
Imaged place cell properties from a representative mouse. **(A)** Linear track with feature-rich contextual cues (textures walls, scored floor, shower curtain design) for the left running direction and feature-space cues (plain walls and shower curtains, smooth floor) for the right running direction. **(B)** Miniature microscope and placement schematic. **(C)** A raw fluorescence field of view (left) and projection map of all cells identified using PCA-ICA (right). Non-place cells (white) and place cells for right (blue) and left running direction (red) derived by spatial information analysis. **(D)** Daily changes in the proportion of place cells within the population of imaged cells superimposed onto the number of linear track runs per imaging session to illustrate correlated behavior and place cell function (Pearson’s *r* = 0.77). The proportion of place cells (linear regression: β = 0.01, *SE* = 0.003, *p* = 0.018) and the number of linear track runs improved significantly with time (linear regression: β = 6.07, *SE* = 1.66, *p* = 0.008). **(E)** Place cell tuning curves from a mouse for left and right running directions, sorted for place field locations during imaging. **(F)** Raw Δ*F*/*F* trace of a single place cell (top panel) and representative firing location from a single place cell during linear track running across multiple recording sessions (bottom panels). Events from a single place cell (filled colored circles) overlaid with mouse position on the linear track (gray dashed lines). Data were acquired at a rate of 10 frames per second. Recording sessions 1–2 were excluded for display purposes. Cort, cortisol; Cit, citalopram.

After training mice to run on the linear track which contained feature-sparse cues (plain walls and shower curtains, smooth floor) for the right running direction and feature-rich contextual cues (textures walls, scored floor, shower curtain design) for the left running direction (Figure 3A), we assessed hippocampal place cell function utilizing miniature fluorescence microscopes (Figure 3B). Thy1-GCaMP6f transgenic mice exhibited a suitable Δ*F*/*F* signal, from which cellular activity could be imaged (Figure 3C, left). Place cells exhibited strong selectivity for running direction (Figure 3C, right). Within each recording session, the cohort of imaged place cells exhibited place-tuned activity patterns that collectively covered the full length of the linear track (Supplementary Figure 2). Place cell coverage of the ends of the linear track, where consumption of the liquid reward occurred, was much denser compared to the center of the linear track. To distinguish spatial coding from reward valence coding, we excluded the two most lateral bins of the linear track from the analysis.

A representative example of long-term stability for CA1 ensembles and single place cells can be seen in Figure 3E,F. The proportion of place cells, defined as the number of place-coding cells divided by the total number of imaged cells, and the number of linear track runs increased significantly with time (Figure 3D). We therefore chose to sort place cells according to their receptive fields on the last recording session to obtain place cell tuning curves with best overall quality. Most place cells were not active during all recording sessions. In untreated mice, the reoccurrence probability of place fields that were active in the first recording session was 39.5% for the second recording session and then gradually dropped to 23.5% by the sixth recording session. To ensure that individual cellular identities were maintained over the duration of the 2-week imaging period, we quantified the degree of pixel shift in the concatenated and aligned calcium imaging data. We found that 90% of displacement errors for registration of cell maps between recording days were smaller than 1.25 microns (data not shown).

### Cortisol Reduces the Proportion of Place Cells

**FIGURE 4:**
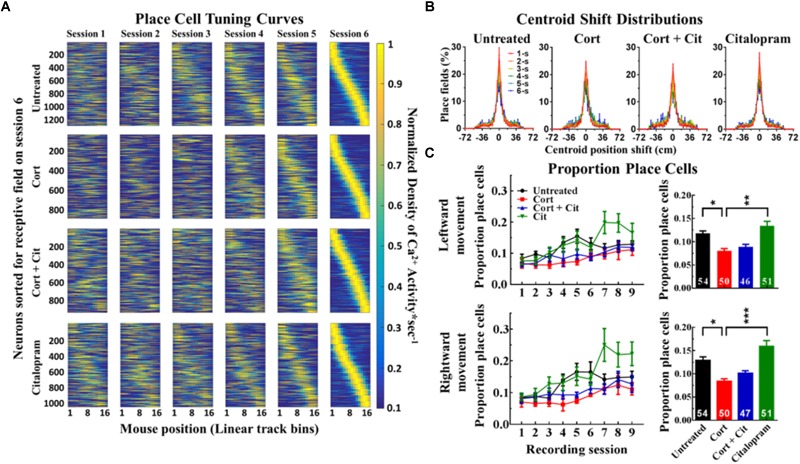
Cortisol treatment induces place cell dysfunction. **(A)** Place cell tuning curves from 24 mice, separated by treatment group (six mice per treatment group), concatenated for left and right running directions. Place cells, identified by shuffle analysis, were sorted by linear track place field location on recording session 6. **(B)** Place field centroid shift distributions between recording session (colored by inter-recording session interval) were indistinguishable within treatment groups (K-S tests, untreated: *p* = 0.58, cortisol: *p* = 0.41, cortisol + citalopram: *p* = 0.27, citalopram: *p* = 0.41) and between groups (*p* = 0.99). Centroid shift distributions peaked at zero centimeters and were distinct from the null hypothesis that place fields would randomly relocate (*p* = 4e^-05^). **(C)** Proportion of identified place cells for individual recording sessions (left) and averaged over all recording sessions (right). Cortisol treatment significantly reduced the proportion of identified place cells as compared to untreated control animals. Cort, cortisol; Cit, citalopram. Asterisks show group comparisons with ^∗^*p* < 0.05, ^∗∗^*p* < 0.01, and ^∗∗∗^*p* < 0.005.

To investigate long-term effects of cortisol treatment on place cell function, we assessed the stability of individual place fields over the course of all imaging sessions for all 24 mice in the four treatment groups (Figure 4A). Place field centroids showed no significant drift between recording sessions, a finding that was consistent across all treatment groups (Figure 4B). Furthermore, there was no significant group difference in place cell stability (Table 1).

To control for potential differences in place cell measures caused by the reduced average number of linear track traversals, and thus reduced sampling information from place cells, in cortisol-treated mice, we limited our place cell analysis to data obtained during the first 50 linear track runs for mice in all treatment groups. We found that cortisol treatment significantly reduced the proportion (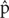) of place cells (left: 0.08 ± 0.005 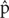; right: 0.084 ± 0.005 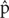) compared to untreated mice (left: 0.118 ± 0.006 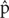; right: 0.129 ± 0.007 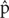) consistently for both feature-rich left-running (β = -0.44, *SE* = 0.16, *p* = 0.03) and for feature-sparse right-running directions (β = -0.48, *SE* = 0.17, *p* = 0.03) after 50 linear track traversals (Figure 4C). Co-administering citalopram with cortisol (left: 0.089 ± 0.006 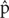; right: 0.101 ± 0.005 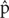) did not rescue the cort-induced reduction in the proportion of place cells. Tukey contrasts for multiple comparisons of means found significant treatment group differences. The proportion of place cells in the cort + cit mice was indistinguishable from mice administered with only cortisol. This was the case for both running directions. Mixed effects models performed for left and right running directions with recording session : treatment groups as fixed factors found that the proportion of place cells increased significantly with recording sessions for all four treatment groups and for both running directions (all comparisons: *p* < 0.001). There was a significant treatment group × recording session interaction effect showing that the proportion of place cells in citalopram-treated mice (left: 0.134 ± 0.01 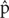; right: 0.159 ± 0.012 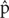) increased significantly more with subsequent recording sessions in comparison to the other treatment groups for both left (β = 0.08, *SE* = 0.027, *p* = 0.003) and right (β = 0.058, *SE* = 0.03, *p* = 0.049) running directions.

Event rate and amplitude, place field size and infield vs. outfield selectivity of firing rate were all indistinguishable between groups (Table 2). There was no significant group difference in the total number of imaged neurons per animal (data not shown). Thus, even though cortisol treatment reduced the number of cells that participate in encoding spatial information, function in place cells spared by cortisol remains intact.

**Table 2:** Place cell properties (mean ± SEM).

	Running direction	Untreated	Cort	Cort + Cit	Citalopram
Proportion place cells ( 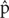 )	Left	0.118 ± 0.006	0.080 ± 0.005^∗^	0.089 ± 0.006	0.134 ± 0.010
	Right	0.129 ± 0.007	0.084 ± 0.005^∗^	0.101 ± 0.005	0.159 ± 0.012
Stability (corr. coef.)	Left	0.76 ± 0.01	0.77 ± 0.01	0.78 ± 0.02	0.79 ± 0.01
	Right	0.77 ± 0.01	0.78 ± 0.01	0.78 ± 0.02	0.81 ± 0.01
Event rate (counts/min)	Left	2.81 ± 0.07	2.28 ± 0.10	2.26 ± 0.08	2.87 ± 0.17
	Right	2.80 ± 0.07	2.22 ± 0.09	2.23 ± 0.09	2.73 ± 0.15
Event amplitude (SD)	Left	5.18 ± 0.04	5.53 ± 0.07	5.56 ± 0.04	5.33 ± 0.07
	Right	5.20 ± 0.05	5.52 ± 0.07	5.58 ± 0.05	5.33 ± 0.06
Place field size (cm)	Left	23.76 ± 1.45	23.36 ± 2.21	23.88 ± 2.36	23.29 ± 1.76
	Right	23.40 ± 1.33	23.30 ± 1.81	23.29 ± 1.79	23.27 ± 1.74
Selectivity (infield/outfield)	Left	0.94 ± 0.02	0.90 ± 0.02	0.88 ± 0.02	0.88 ± 0.02
	Right	0.96 ± 0.02	0.90 ± 0.02	0.91 ± 0.02	0.92 ± 0.02

*Summary table detailing all assessed place cell properties from untreated, cort-treated, cort + cit treated and citalopram-treated mice. Properties for each group are separated into left and right running directions. Values reported as mean ± SEM. ^∗^*p* < 0.05.*

### Cortisol Treatment Increases Single Neuron Decoder Prediction Error

**FIGURE 5:**
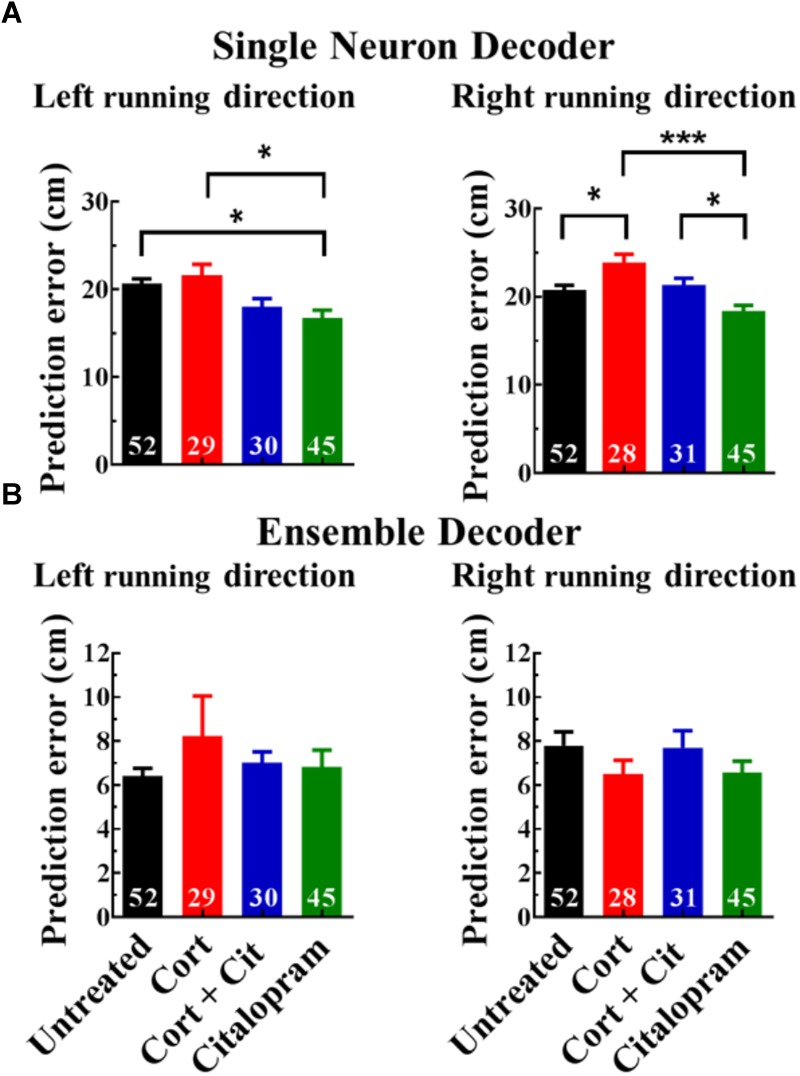
Cortisol treatment increases the single neuron prediction error. **(A)** Bar graphs of single neuron mouse location prediction errors for all neurons (457 ± 25 cells per group) averaged over all recording sessions (8.58 ± 0.16 sessions) [mean ± SEM]. Calculations were averaged by mice (*n* = 6) per group for left and right running directions separately. Cortisol-treated mice had a significantly higher prediction error (*p* < 0.05) compared to untreated mice for the rightward running direction only, but a higher prediction error for both running directions when compared to citalopram-treated mice. Asterisks show group comparisons with ^∗^*p* < 0.05 and ^∗∗∗^*p* < 0.005. **(B)** Mouse location prediction errors using Bayesian decoders trained on ensemble neural data. No significant group effects were found for the ensemble decoders. Data shown as mean ± SEM, (*n* = 6 mice).

To further investigate the effect of the reduced number of place cells in cortisol-treated mice, we trained decoders using calcium imaging data to predict mouse position on the linear track (see section “Materials and Methods”). We first trained a single neuron decoder that predicted the position of the mouse from the activity of just one neuron. To circumvent differences in data volume between groups for the decoder performance, we predicted mouse position by randomly selecting 20 runs 10 times to train single neuron Bayesian decoders. A mixed effects estimation with neuron effects nested in mouse effects found that cortisol-treated mice showed a significantly higher prediction error (23.70 ± 1.13 cm) [mean ± SEM] for the feature-sparse, right running direction (β = 3.01, SE = 1.52, *p* < 0.05 cm) compared to untreated mice (20.58 ± 0.75 cm), whereas the feature-rich leftward running direction did not yield significant cortisol treatment effect between untreated (20.69 ± 0.52 cm) and cortisol-treated mice (21.64 ± 1.21 cm) (Figure 5A). A mixed effects model found that the prediction error in cortisol-treated mice was consistently higher compared to the citalopram group for both running directions (left: 16.74 ± 0.89 cm, β = 5.12, *SE* = 2.05, *p* < 0.01; right: 18.16 ± 0.87 cm, β = 5.40, *SE* = 1.55, *p* = 0.0005). As with previous place cell measures, mixed effects models showed that co-administration of citalopram with cort did not improve the higher prediction error in cortisol-treated mice, as there were no significant group differences for either running direction.

To determine if the degraded representation of mouse position at the single neuron level in mice treated with cortisol was also present at the ensemble level, we also trained decoders to predict mouse track position from the entire population of neurons. The ensemble decoder computed the median absolute decoder error on a held-out portion of the dataset for a given recording session, as used in Ziv et al. (2013). For this decoder, mouse position was determined with the held-out data from the same day on which the decoder was trained (Figure 5B). For the left running direction, prediction errors of untreated mice (6.41 ± 0.35 cm), cort-treated mice (8.23 ± 1.82 cm), cort + cit-treated mice (7.01 ± 0.51 cm) and cit-treated mice (6.82 ± 0.78 cm) did not produce significantly different mixed effects between treatment groups. The same was true for the right running direction, with similar prediction errors (untreated: 7.80 ± 0.63 cm, cort: 6.50 ± 0.64 cm, cort + cit: 7.70 ± 0.78 cm, cit: 6.57 ± 0.53 cm).

## Discussion

The goal of this study was to create a mouse model of chronic stress in which the neural effects of stress, and treatment with citalopram, could be assessed during behavior. There is a rich body of literature in which chronic stress has been modeled by administering corticosteroids in the drinking water or by subcutaneous injection, with doses and durations of administration ranging widely (Sapolsky et al., 1985; Bodnoff et al., 1995; Magarinos et al., 1998, 1999; Sousa et al., 1998a,b; Gourley et al., 2008; Mitra and Sapolsky, 2008). The most widely used dose is around 35 μg/ml, which is close to the dose of 40 μg/ml used in the current study. The administration regimen employed in this study was sufficient to produce physiological effects and behavioral symptoms of chronic stress. The weight of the adrenal glands, the corticosterone producing organs, was robustly affected by cortisol administration, consistent with increased exogenous cortisol levels negatively regulating the production of endogenous corticosterone (Young et al., 1995). Chronic stress also suppresses immune system function (Glaser and Kiecolt-Glaser, 2005). Along these lines, spleen weight was reduced in our study in mice administered with cortisol. Cortisol-treated mice showed a lack of preference for sniffing urine over water in the FUST, a model of anhedonia, a criterion for major depressive disorder. Furthermore, cortisol-treated mice showed a reduction in reward seeking behavior during linear track running, as demonstrated by reduced liquid reward consumption on the linear track and significantly slower running speeds to obtain these rewards. Taken together, these behavioral findings are similar to symptoms found in patients with major depressive disorder (American Psychiatric Association, 2013).

The effects of stress on hippocampal functions are complex and heavily dependent on stress-to-task latency, exposure and duration. For example, a transient stressor is sufficient to affect risk-taking behavior (Porcelli and Delgado, 2017), whereas a prolonged chronic stressor causes morphological and neurodegenerative symptoms that impair learning, memory and spatial navigation (Kim and Yoon, 1998; Kim et al., 2015). An elegant method to investigate how stress contributes to cognitive deficits is to study processing of spatial information by hippocampal place cells in stressed rodents during spatial navigation and memory formation. There have been several pioneering studies that have begun to address this question. For example, Kim et al. (2007) showed that 2 h of acute audiogenic stress causes deficits in place cell activity during open field exposure, as electrophysiological recordings from the dorsal hippocampus revealed that stress impaired the stability of place cell firing rates. The authors suggest that stress-induced modifications in synaptic plasticity may prevent the long-term stability of place cell tuning curves, which could contribute to spatial memory deficits. Similarly, 30 min of acute photic stress can impair spatial information processing of place cells. Passecker et al. (2011) showed that a photic stressor significantly decreases CA1 and CA3 place cell firing frequencies and overall burst activity. However, the acute stressor spared spatial location characteristics of place cells, such as spatial information content and spatial selectivity. Park et al. (2015) and Tomar et al. (2015) explored the effects of prolonged and repeated immobilization stress on the physiology of hippocampal place cells in mice. Tomar et al. (2015) found that a shorter duration of 5 days of immobilization stress results in decreased excitability of CA1 pyramidal cells, whereas 11 days of immobilization impaired context discrimination, suggesting that a loss of network flexibility may underlie some of the behavioral deficits accompanying prolonged stress. Similarly, Park et al. (2015) restrained mice for 21 days and found that chronic stress modifies physical properties of place cell spiking during spatial navigation, such as decreased stability of spatial representation and firing rates. Thus, next to its effect as a powerful modulator of mood and motivation, chronic stress can have a significant impact on hippocampal function and pathophysiology.

One of the main findings of this study was that chronic cortisol treatment reduced the proportion of place cells amongst the population of imaged neurons in CA1. This effect was highly consistent for both left and right running directions. This novel finding was enabled by the use of miniature microendoscopy, which allows for the interrogation of neural function at the ensemble level, as hundreds of neurons were imaged simultaneously without bias for place cells over non-place cells. Surprisingly, functional properties of the remaining place cells were unaffected after cortisol treatment. This could indicate that stress may reduce the number of neurons that can be recruited for processing information during spatial navigation, whereas neurons spared by cortisol treatment could function without perturbation.

Since cortisol-treated mice exhibited fewer runs per imaging session on the linear track and ran at a slower speed, we wanted to rule out that group differences in data quality and volume affected our place cell measures. To do so, we limited our analysis to the first 50 linear track runs. The overall proportion of identified place cells was lower after 50 runs compared to all runs, however, the general group differences at 50 runs were comparable to all runs (data not shown), suggesting that 50 runs are sufficient to estimate treatment group differences. When only using a single neuron to predict mouse position, we found that the prediction error was significantly higher in cortisol-treated mice, but only for rightward movement. One possible explanation for the differences in left vs. right movement is that spatial encoding of information is facilitated by spatial cues, as we designed the left half of the linear track with rich spatial cues and the right half with poor spatial cues. There were no significant treatment group effects for the ensemble decoder prediction error of mouse position. The dysfunction observed at the single-neuron level indicates that chronic stress may impair the ability of the hippocampus to encode neural representations of the mouse’s spatial position, a function pivotal for forming or maintaining an accurate navigational map of the mouse’s external environment. The results from the ensemble decoders support the finding that the hippocampal ensemble, as a whole, may be resilient to any cortisol-induced insults to single neuronal place cell function on the linear track.

Citalopram has been reported to alleviate behavioral symptoms of chronic stress related to motivation and reward seeking behavior (Rygula et al., 2006; Kusmider et al., 2007; Ara and Bano, 2012; Araya-Callis et al., 2012; Chen et al., 2012). The administered dose of 10 mg/kg citalopram used in this study has been shown to reliably increase serum and brain concentrations (Karlsson et al., 2013a,b) and falls well within the therapeutic dose to alleviate symptoms of depression. Citalopram improved the single neuron decoder prediction performance compared to untreated and cortisol-treated mice but failed to rescue other deficits in place cell function induced by chronic cortisol treatment. Additionally, citalopram did not ameliorate the depression-like symptoms exhibited in the FUST nor the reduction in linear track running seen in the cortisol-treated animals. We did not expect citalopram to prevent the reduction in organ weights, since exogenous administration of cortisol down-regulates endogenous production of corticosterone and therefore leads to small adrenals weight. One possible explanation of the inability of citalopram to rescue the phenotypes of stress and depression-like behavior in this study could be the limitation of the experimental design. It was necessary to water restrict mice to train them to run back and forth a linear track to retrieve water rewards; however, water restriction itself is a stressor (Wotus and Engeland, 2003). When combined with cortisol administration, the cumulative amount of induced stress might have been too severe for citalopram to compensate. Prior studies demonstrating citalopram’s effectiveness in alleviating symptoms of stress only had one stressor present. When multiple stressors impinge on mice, however, paradoxical results can be observed (Magarinos et al., 1998).

The novel contributions made by this study are the unbiased, long-term recording of hundreds of place cells exposed to chronic corticosteroids during linear track running, and the investigation of the effects of the stress-reducing antidepressant citalopram on place cell function under normal and conditions of corticosteroid administration. In conclusion, cortisol induced physiological and behavioral changes consistent with effects observed in models of chronic stress. The overall proportion of hippocampal place cells encoding spatial location was reduced in a way that was irreversible by citalopram administration; however, the hippocampus also displays a considerable robustness to insults as shown by the intact ensemble decoder performance.

## Data Availability

The raw data supporting the conclusions of this manuscript will be made available by the authors, without undue reservation, to any qualified researcher. The datasets generated for this study are available on request to the corresponding author.

## Author Contributions

TI, MS, JN, PB, and RW designed the experiments. TI and NW performed the experiments. TI, MS, SY, JN, and RW analyzed the data. TI, MS, JN, PB, and RW wrote the manuscript. TI, MS, SY, SO, JN, TL, PB, and RW contributed tools, materials, and expertise. SO, TL, and PB supervised the study.

## Conflict of Interest Statement

TI, SY, NW, TL, PB, and RW are paid employees at Janssen Pharmaceutical Research & Development, LLC. MS, SO, and JN are paid employees at Inscopix. TI was a paid employee at Janssen Research & Development while the experimental work was performed and was employed by AnaBios Corporation during the time that the manuscript was submitted to Frontiers in Neuroscience. AnaBios Corporation had no competing interests. The authors maintain adherence to the journal’s policies on sharing data and materials.
