# Supplementary material for: In vivo Calcium Imaging Reveals That Cortisol Treatment Reduces the Number of Place Cells in Thy1-GCaMP6f Transgenic Mice
Source: Front Neurosci. 2019 Mar 1;13:176. doi: 10.3389/fnins.2019.00176 (PMC6405689; doi:10.3389/fnins.2019.00176)
Supplement: Supplementary file 1 [file Data_Sheet_1.pdf]

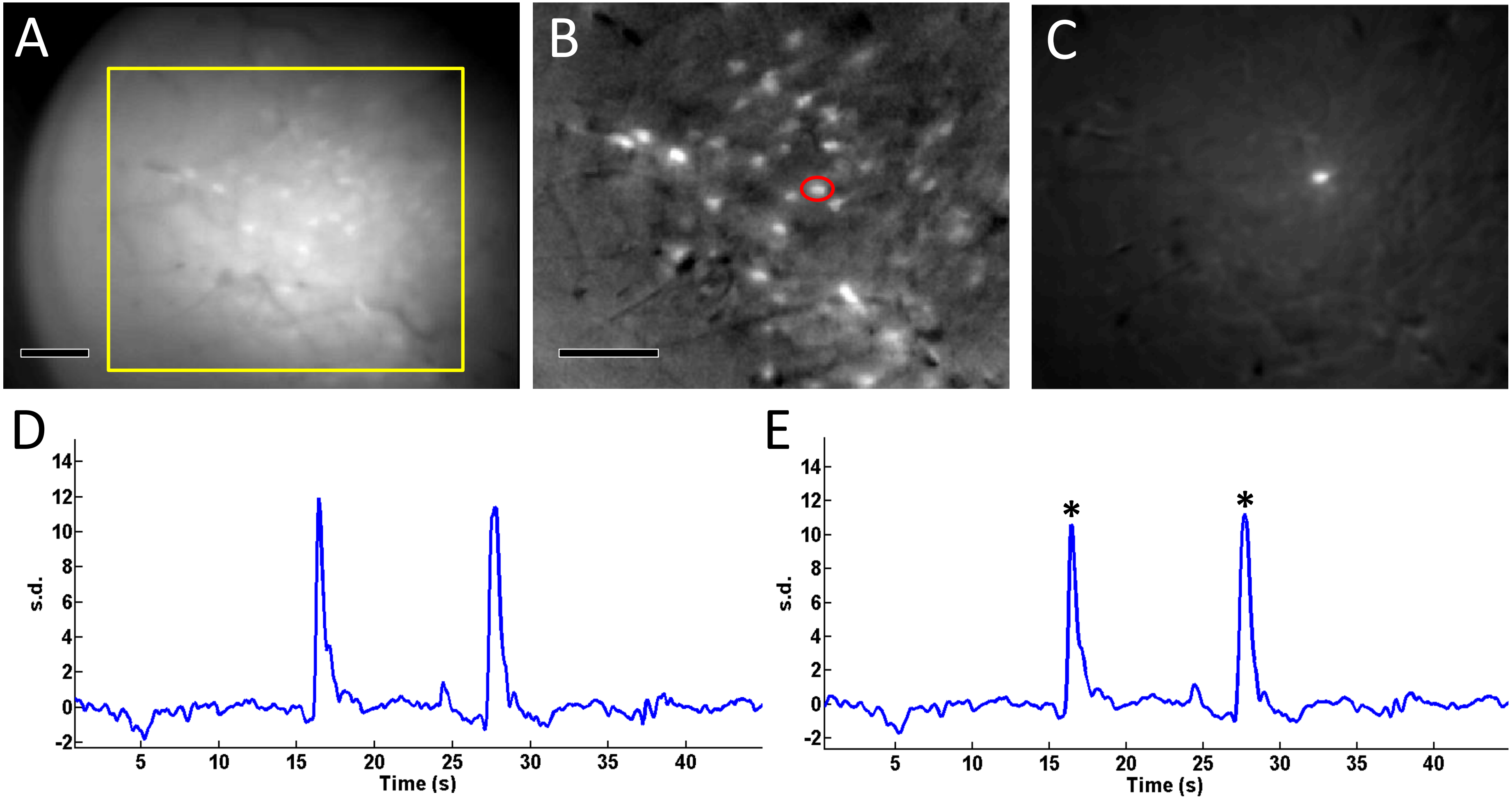

Supplementary Figure 1. Calcium imaging data processing.

(A) Raw fluorescence image of active GCaMP6f-expressing neurons in CA1 taken from a movie acquired while a mouse was running on the linear track. **(B)** Spatially down-sampled and ΔF/F-transformed image of boxed area in A. Transformation was applied to all frames in the movie. **(C)** Isolated cell (red circle in B) identified via PCA/ICA processing of ΔF/F-transformed movie. **(D)** Trace illustrating relative fluorescence changes (standard deviations, s.d.) of the identified cell. **(E)** 1 Hz-filtered trace with asterisks identifying detected calcium events. Scale bars: 100 μm.

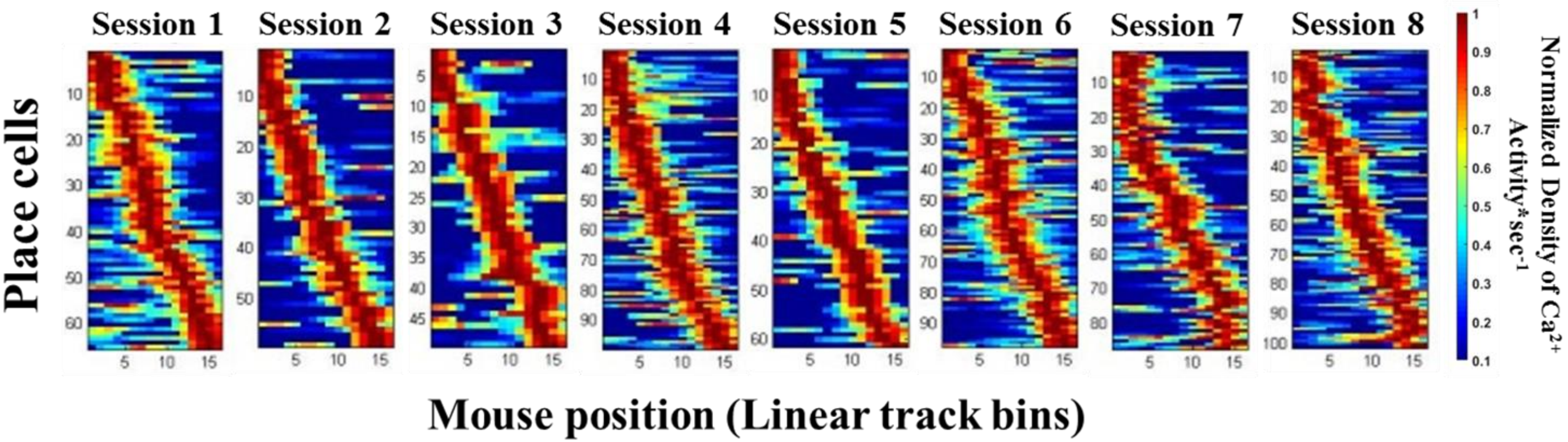

Supplementary Figure 2. Within-day single animal place cell tuning curves. Place cells identified by the shuffle analysis sorted with respect to their place field locations on the linear track for that day.
